# The effect of CPAP therapy on heart rate variability in patients with obstructive sleep apnea

**DOI:** 10.1007/s41105-022-00424-2

**Published:** 2022-09-14

**Authors:** Magda Grzęda-Hałon, Małgorzata Poręba, Gabriela Gut, Karolina Czerwińska, Paweł Gać, Helena Martynowicz, Grzegorz Mazur, Rafał Poręba

**Affiliations:** 1 Department of Internal Medicine, Occupational Diseases and Hypertension, Wroclaw Medical University, Borowska 213, 50-556 Wroclaw, PL Poland; 2 Department of Paralympic Sports, Wroclaw University of Health and Sport Sciences, Witelona 25a, 51-617 Wroclaw, PL Poland; 3 Department of Population Health, Division of Environmental Health and Occupational Medicine, Wroclaw Medical University, Mikulicza-Radeckiego 7, 50-368 Wroclaw, PL Poland

**Keywords:** CPAP, Heart rate variability, Obstructive sleep apnea

## Abstract

The aim of this study was to analyze the relationship between the initiation of CPAP therapy and HRV in patients with OSA. The study group consisted of 37 patients, aged 34–79 (mean 54.95 years) with OSA treated with CPAP. Two subgroups of patients were distinguished: less than severe (AHI < 30, *n* = 16) and severe OSA (AHI ≥ 30, *n* = 21). The second study was carried out around a month after the initiation of therapy. CPAP therapy caused the improvement in polysomnographic parameters, however, in most parameters in time and frequency analysis, there were no significant positive changes in parasympathetic tone. Moreover, in HRV time analysis, the reduced rMSSD and pNN50 parameters in the hours of night rest and rMSSD and SDSD during the 15-min N3 sleep period were noted. Especially, in the group with AHI ≥ 30, we observed significant decreases in rMSSD and pNN50 for the entire time. The changes were mainly for the night periods including the N3 sleep period, which is especially connected with sleep apnea (parameters: rMSSD, SDSD, and pNN50). In spectral analysis, the decrease in HF from the 15-min daily activity period and the N3 sleep period was observed. Inverse correlations were seen between the maximum, median, and mean positive airway pressure (PAP) and the change in rMSSD, SDNN, and SDSD, mainly during night hours and the N3 sleep period. Only in patients with AHI < 30 the increase in SDNN was observed in 15-min N3 sleep period. The beneficial increase in SDNN parameter from time analysis was observed only in one sleep period in less ill patients with OSA. The lack of significant changes was observed in the majority of the parameters of heart rate variability after initiation of CPAP therapy in a short observational time; however, the shift towards reduced HRV was observed in patients with AHI > 30, so the response to CPAP therapy may depends on the severity of the apnea. The results may suggest that a longer observational period is needed in such studies, and the problem is still not fully elucidated.

## Introduction

Obstructive sleep apnea (OSA) is a common breathing disorder diagnosed when at least 5 such apnea and/or hypopnea episodes are detected [1].

It is estimated that this problem affects approximately 4% of men and 2% of women, however, some studies suggest that milder forms of OSA can be diagnosed even in 38% of adults.

Sleep-related breathing disorders lead to hypoxia, disturbances in sleep patterns, acceleration of heart rate and increase in blood pressure. This further contributes to cardiovascular diseases such as arterial hypertension, ischemic heart disease, stroke, as well as arrhythmias, in particular night arrhythmias [2]

The CPAP therapy is a gold standard in the treatment of OSA. It prevents the upper airway from collapsing and keeps it open through the positive pressure generated by the device [3].

Heart rate variability (HRV) analysis, calculated from the 24-h Holter ECG examination, is considered a simple, non-invasive way to test the efficiency of the autonomic nervous system [4, 5]. In patients with untreated OSA, the function of the autonomic nervous system is often impaired [6, 7]. These patients exhibit a reduction in vagal tone along with an increased response of the sympathetic nervous system, which causes an imbalance between the parasympathetic and sympathetic systems [8].

Activation of the sympathetic system in patients with OSA, mainly due to recurrent obstruction of the upper respiratory tract, is one of the main mechanisms of the development of cardiovascular diseases [4, 8].

It has been reported that CPAP therapy can reduce HRV during sleep and decrease the activity of the sympathetic nervous system [9].

This study aimed to analyze the relationship between the initiation of CPAP therapy and HRV in patients with OSA.

## Materials and methods

All participants were hospitalized at the Department of Internal and Occupational Diseases, Hypertension and Clinical Oncology in Wrocław. The study group consisted of 37 patients, 28 men and 9 women aged 34–79 (mean 54.95 years), all with moderate or severe OSA, qualified for CPAP therapy. Participants were assigned to the CPAP treatment and were connected with the auto-adjusting CPAP machine (Autoset S8, ResMed, Abingdon, UK). Each patient underwent two full unsupervised polysomnography (type II) tests using ResMed's Nox1A1 apparatus. Both polysomnograms included the following: electroencephalogram (EEG), electrooculogram (EOG), electromyogram (EMG) of the chin and lower limbs, digital pulse oximetry, electrocardiogram (ECG), oral and nasal airflow temperature and pressure registration using a thermistor and pressure sensor and recording of respiratory effort using induction plethysmography. Most of the qualified patients suffered from obesity (67.6%), hyperlipidemia (75.7%) and hypertension (67.6%). Moreover, 27% were diagnosed with diabetes, 10.8% with ischemic heart disease and 5.4% had a history of stroke. Among the patients, 18.9% declared active smoking. The exclusion criterion was atrial fibrillation and pauses longer than 2.5 s recorded during the Holter examination.

Based on the severity of OSA, two subgroups of patients were distinguished: patients with OSA less than severe (AHI before the initiation of CPAP therapy < 30, *n* = 16) and with severe OSA (AHI before the initiation of CPAP therapy ≥ 30, *n* = 21). The characteristics of the study group are presented in Table 1.

**Table 1:** Clinical characteristics of the study group (*n* = 37)

	Mean	Median	Minimum	Maximum	SD
Age (years)	54.95	58.00	34.00	79.00	11.86
Hight (meters)	1.72	1.72	1.58	1.91	0.08
Body weight (kg)	101.55	100.00	62.00	134.00	18.29
BMI (kg/m^2^)	33.96	33.69	24.22	47.27	5.40
Glucose (mg/dl)	122.74	108.00	89.00	257.00	40.34
Total cholesterol (mg/dl)	219.93	225.50	140.00	298.00	49.93
HDL cholesterol (mg/dl)	48.14	46.00	33.00	77.00	10.68
LDL cholesterol (mg/dl)	130.82	134.00	53.00	195.00	43.51
Triglycerides (mg/dl)	204.75	195.00	86.00	371.00	68.70
Mean PAP (cmH2O)	5.97	5.10	– 1.00	16.70	3.43
Maximum PAP (cmH2O)	13.32	12.60	– 1.00	45.40	8.44
Median PAP (cmH2O)	5.85	5.10	– 1.00	16.90	3.62
PAP 95%	8.58	8.50	– 1.00	19.00	4.53

	*n*		%		
Men	28		75.7		
Women	9		24.3		
Obesity	25		67.6		
Diabetes	10		27.0		
Hyperlipidaemia	28		75.7		
Arterial hypertension	25		67.6		
Ischemic heart disease	4		10.8		
Stroke	2		5.4		
Smoking	7		18.9		

All patients completed the proprietary questionnaire, which included questions about age, sex, body weight, height, cardiovascular diseases and cardiovascular risk factors. Then, the participants underwent laboratory tests, including HDL cholesterol, LDL cholesterol, total cholesterol and triglycerides levels. Later in the study, each patient had two 24-h ECG recordings:

- the first record—before the initiation of CPAP therapy,
- the second record—after the initiation of CPAP therapy.

The second recording was carried out about one month after introducing CPAP therapy.

Heart rate variability (HRV) was assessed using time analysis and spectral/frequency analysis during a 24-h Holter analysis using the Pathfinder system (Spacelabs Healthcare, Hertford, UK) [2].

The time analysis was performed by statistical methods using parameters such as mRR—mean RR interval during sinus rhythm, SDNN—standard deviation of all NN intervals, rMSSD—the square root of the mean of the sum of the squares of differences between adjacent NN intervals, SDSD—standard deviation of differences between adjacent NN intervals and pNN50—NN50 count divided by the total number of all NN intervals. The parameters were analyzed for the entire day and separately for the hours of daily activity and night rest. The 15-min periods during daytime activity and the N3 sleep phase were also analyzed.

The spectral analysis was performed using the Fast Fourier Transform (FFT). It included such parameters as VLF—the power of very low-frequency spectrum (0.003–0.04 Hz), LF—the power of low-frequency spectrum (0.04–0.15 Hz), HF—the power of high-frequency spectrum (0.15–0.4 Hz), VHF—the power of very high-frequency spectrum (0.4–0.9 Hz), LF/HF—a ratio of powers LF/HF. These parameters were analyzed for the entire study period and separately for the hours of daily activity and night rest, as well as for the 15-min daily activity period and the N3 sleep period.

**Fig. 1:**
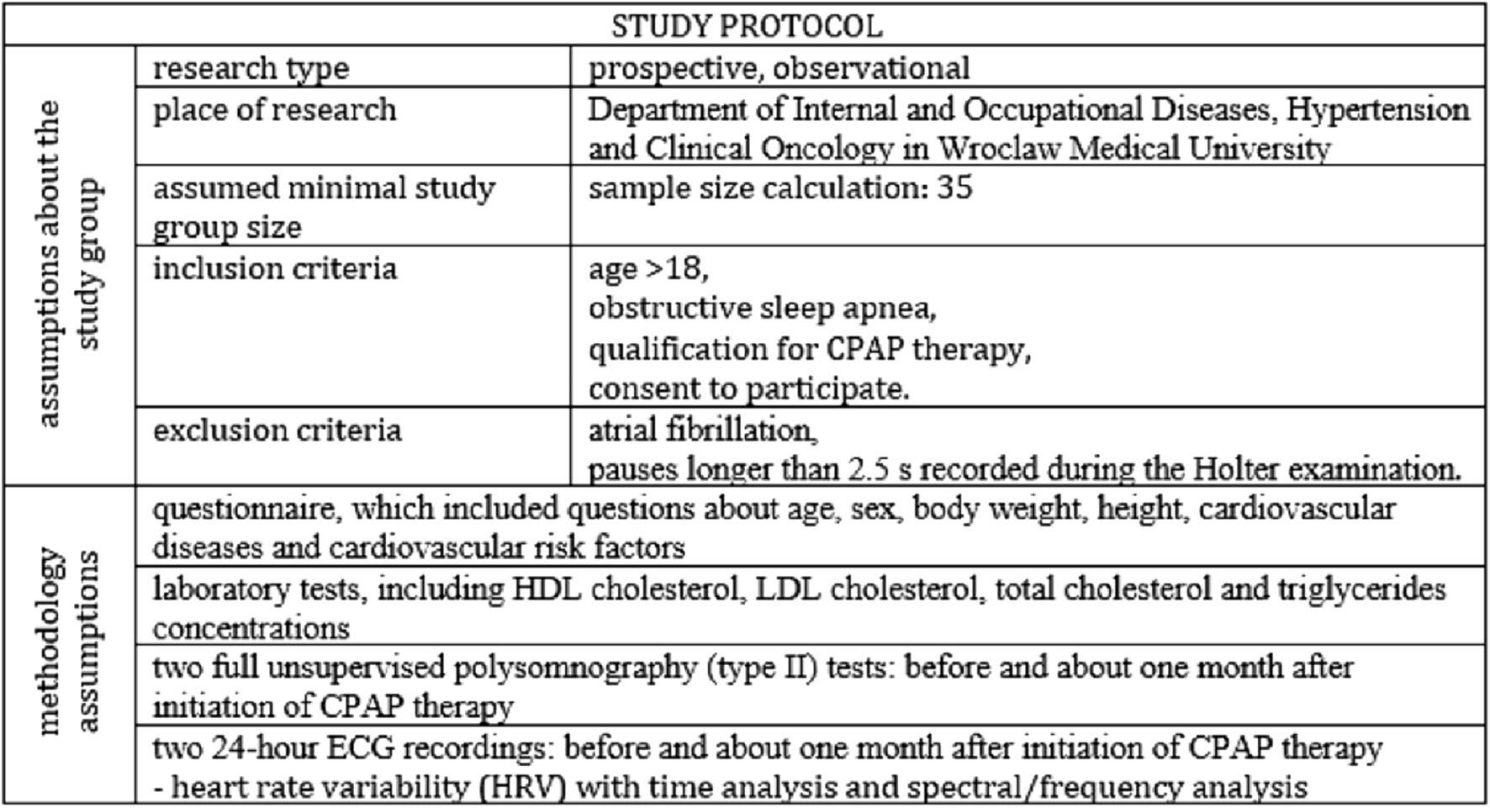
Flowchart of the study protocol

The flowchart of the study protocol is shown in Fig. 1.

Statistical analysis was performed using the Dell Statistica 13 (Dell Inc., Tulsa, OK, USA) application. For quantitative variables, arithmetic means and standard deviations were calculated. The Shapiro–Wilk test was used to verify the normal distribution of the variables. Quantitative independent variables with normal distribution were further analyzed using a t-test for independent variables. Variables with distribution other than normal were analyzed using the U Mann–Whitney test for independent quantitative variables. For the dependent quantitative variables of the normal distribution, the t-test for linked variables was used. In cases of quantitative dependent variables showing the distribution distinct from normal, the pair sequence test of Wilcoxon was applied. The correlation was analyzed to specify the relationship between the analyzed variables. Pearson correlation coefficients were determined for quantitative variables with normal distribution, and Spearman correlation coefficients for quantitative variables with distribution other than normal. The adopted statistical significance level was *p* < 0.05.

## Results

Initiation of CPAP therapy resulted in the following changes in polysomnographic parameters: statistically significantly lower AHI, greater sleep latency, lower sleep efficiency, lower oxygen desaturation index (ODI), higher mean and minimum SpO2, and a shorter% of time with SpO2 < 90%. Selected polysomnographic parameters before and after the initiation of CPAP are summarized in Table 2.

**Table 2:** Selected polysomnographic parameters before and after the initiation of CPAP therapy (*n* = 37)

	Before the initiation of CPAP	After the initiation of CPAP	*p*
AHI	41.95 ± 24.44	7.58 ± 11.48	< 0.05
Sleep latency	16.24 ± 13.35	37.76 ± 47.03	< 0.05
Sleep efficiency	85.18 ± 7.36	72.57 ± 19.22	< 0.05
NREM 1 (%)	11.49 ± 9.96	8.63 ± 7.96	ns
NREM 2 (%)	49.62 ± 11.88	49.63 ± 14.37	ns
NREM 3 (%)	16.69 ± 8.75	18.64 ± 12.25	ns
REM (%)	22.22 ± 8.55	23.11 ± 9.63	ns
ODI	41.96 ± 23.73	13.54 ± 17.22	< 0.05
Mean SpO2	90.68 ± 2.79	92.91 ± 2.13	< 0.05
Minimum SpO2	76.00 ± 8.99	85.24 ± 6.94	< 0.05
% of the SpO2 < 90% time	26.15 ± 25.22	8.53 ± 15.35	< 0.05

In terms of the HRV time analysis, it was shown that CPAP therapy reduced rMSSD and pNN50 in the hours of night rest and rMSSD and SDSD during the 15-min N3 sleep period. The time analysis of HRV parameters before and after the CPAP therapy is presented in Table 3.

**Table 3:** Parameters of heart rate variability time analysis before and after the initiation of CPAP therapy (*n* = 37)

	Before the initiation of CPAP	After the initiation of CPAP	*p*
Entire day (6:00–6:00)			
mRR (ms)	887.71 ± 111.52	894.19 ± 122.40	ns
SDNN (ms)	115.14 ± 34.20	116.05 ± 40.76	ns
rMSSD (ms)	34.46 ± 21.33	26.98 ± 10.55	ns
SDSD (ms)	26.94 ± 21.01	19.91 ± 8.67	ns
pNN50 (%)	7.77 ± 7.40	5.87 ± 6.32	ns
Hours of daily activity (6:00–22:00)			
mRR (ms)	848.79 ± 117.72	849.83 ± 122.57	ns
SDNN (ms)	103.39 ± 31.33	97.80 ± 31.51	ns
rMSSD (ms)	30.09 ± 20.19	24.44 ± 9.35	ns
SDSD (ms)	23.50 ± 19.66	17.98 ± 7.54	ns
pNN50 (%)	5.52 ± 6.89	4.59 ± 5.65	ns
Hours of night rest (22:00–6:00)			
mRR (ms)	954.46 ± 110,72	970.69 ± 135.54	ns
SDNN (ms)	93.26 ± 28.40	91.99 ± 33.47	ns
rMSSD (ms)	39.68 ± 23.99	30.41 ± 13.56	*p* < 0.05
SDSD (ms)	29.93 ± 23.38	21.79 ± 11.02	ns
pNN50 (%)	11.32 ± 10.26	8.08 ± 8.48	*p* < 0.05
15-min daily activity period			
mRR (ms)	853.32 ± 143.94	846.79 ± 157.53	ns
SDNN (ms)	60.14 ± 28.13	53.31 ± 24.87	ns
rMSSD (ms)	27.26 ± 21.88	21.50 ± 9.76	ns
SDSD (ms)	17.04 ± 8.61	14.64 ± 6.81	ns
pNN50 (%)	7.38 ± 16.81	3.85 ± 6.07	ns
15-min N3 sleep period			
mRR (ms)	962.29 ± 131.45	978.86 ± 140.90	ns
SDNN (ms)	54.99 ± 24.70	57.46 ± 38.09	ns
rMSSD (ms)	38.61 ± 21.56	29.49 ± 14.18	< 0.05
SDSD (ms)	27.75 ± 20.12	20.28 ± 10.77	< 0.05
pNN50 (%)	11.60 ± 13.33	8.24 ± 9.62	ns

The HRV spectral analysis did not show any differences between the ECG recording performed before and after the initiation of CPAP. The full results of the spectral analysis are presented in Table 4.

**Table 4.** Parameters of heart rate variability spectral (frequency) analysis before and after the initiation of CPAP therapy (*n* = 37)

	Before the initiation of CPAP	After the initiation of CPAP	*p*
Entire day (6:00–6:00)			
VLF (ms^2^)	10,265.15 ± 55,110.67	858.72 ± 658.08	ns
LF (ms^2^)	1474.42 ± 4732.60	726.69 ± 946.66	ns
HF (ms^2^)	535.32 ± 834.57	324.56 ± 358.00	ns
VHF (ms^2^)	73.79 ± 80.69	61.82 ± 115.85	ns
LF/HF	2.20 ± 1.34	2.60 ± 2.15	ns
15-min daily activity period			
VLF (ms^2^)	807.87 ± 1090.77	554.96 ± 636.11	ns
LF (ms^2^)	403.38 ± 394.86	360.47 ± 406.50	ns
HF (ms^2^)	194.24 ± 185.86	139.32 ± 168.38	ns
VHF (ms^2^)	39.68 ± 41.56	29.71 ± 32.13	ns
LF/HF	3.05 ± 2.43	4.30 ± 3.68	ns
15-min N3 sleep period			
VLF (ms^2^)	1850.81 ± 5180.42	780.13 ± 800.52	ns
LF (ms^2^)	912.78 ± 2365.24	641.48 ± 938.22	ns
HF (ms^2^)	567.24 ± 748.48	351.31 ± 430.61	ns
VHF (ms^2^)	66.04 ± 63.85	49.26 ± 79.68	ns
LF/HF	3.33 ± 10.38	3.44 ± 4.70	ns

Tables 5 and 6 present a comparative analysis of the time and spectral HRV analysis in the subgroups differing in the severity of OSA. Interestingly, in terms of spectral analysis, in both subgroups, i.e., with AHI above and below 30, no significant changes were observed between the first and second ECG recording. On the other hand, the time analysis showed significant changes within the pNN50 parameter in the 24-h and, separately, day and night measurements, as well as in the 15-min N3 sleep period. It is worth noting that in the group of severely ill patients (AHI ≥ 30), this was a significant decrease in the value, and for the rMSSD parameter, such a significant decrease was observed at night.

**Table 5:** Change in the parameters of heart rate variability time analysis in subgroups differing in the severity of obstructive sleep apnea (delta means the difference between the first and second measurement)

	AHI before the initiation of CPAP < 30 (*n* = 16)	AHI before the initiation of CPAP ≥ 30 (*n* = 21)	*p*
Entire day (6:00–6:00)			
Δ mRR (ms)	43.65 ± 79.71	− 21.84 ± 119.17	ns
Δ SDNN (ms)	4.73 ± 25.84	− 2.00 ± 35.29	ns
Δ rMSSD (ms)	0.12 ± 10.48	− 13.27 ± 27.87	ns
Δ SDSD (ms)	− 0.96 ± 9.35	− 11.66 ± 27.21	ns
Δ pNN50 (%)	1.55 ± 5.16	− 4.54 ± 8.21	< 0.05
Hours of daily activity (6:00–22:00)			
Δ mRR (ms)	33.40 ± 79.88	− 23.63 ± 122.30	ns
Δ SDNN (ms)	− 3.59 ± 22.48	− 7.11 ± 32.13	ns
Δ rMSSD (ms)	0.16 ± 10.09	− 10.08 ± 26.51	ns
Δ SDSD (ms)	− 1.23 ± 8.77	− 8.78 ± 25.75	ns
Δ pNN50 (%)	1.79 ± 4.47	− 3.01 ± 8.09	< 0.05
Hours of night rest (22:00–6:00)			
Δ mRR (ms)	57.60 ± 94,88	− 15.29 ± 125.40	ns
Δ SDNN (ms)	6.89 ± 17.19	− 7.49 ± 30.51	ns
Δ rMSSD (ms)	0.45 ± 11.74	− 16.68 ± 30.48	< 0.05
Δ SDSD (ms)	− 0.09 ± 10.77	− 14.26 ± 29.96	ns
Δ pNN50 (%)	1.30 ± 7.23	− 6.70 ± 9.41	< 0.05
15-min daily activity period			
Δ mRR (ms)	34.91 ± 129,05	− 38.10 ± 190.68	ns
Δ SDNN (ms)	− 3.94 ± 24.50	− 9.03 ± 36.91	ns
Δ rMSSD (ms)	2.29 ± 8.73	− 11.90 ± 30.45	ns
Δ SDSD (ms)	1.21 ± 5.90	− 5.16 ± 11.45	< 0.05
Δ pNN50 (%)	2.53 ± 6.74	− 8.15 ± 22.95	ns
15-min N3 sleep period			
Δ mRR (ms)	50.97 ± 106.14	− 9.65 ± 118.14	ns
Δ SDNN (ms)	18.42 ± 48.66	− 9.68 ± 38.92	ns
Δ rMSSD (ms)	− 1.86 ± 18.34	− 14.65 ± 21.61	ns
Δ SDSD (ms)	− 2.19 ± 17.59	− 11.49 ± 21.36	ns
Δ pNN50 (%)	0.42 ± 9.10	− 6.23 ± 10.32	< 0.05

Δ = measurement after the initiation of CPAP—measurement before the initiation of CPAP (positive values—parameter increase, negative values—parameter decrease)

**Table 6:** Change in the parameters of heart rate variability spectral analysis in subgroups differing in the severity of obstructive sleep apnea (delta means the difference between the first and second measurement)

	AHI before the initiation of CPAP < 30 (*n* = 16)	AHI after the initiation of CPAP ≥ 30 (*n* = 21)	*p*
Entire day (6:00–6:00)			
Δ VLF (ms^2^)	− 138.32 ± 458.04	− 16,467.85 ± 73,283.33	ns
Δ LF (ms^2^)	− 54.61 ± 377.88	− 1275.82 ± 6458.42	ns
Δ HF (ms^2^)	− 22.04 ± 276.15	− 354.54 ± 1128.04	ns
Δ VHF (ms^2^)	− 10.90 ± 70.93	− 12.79 ± 98.79	ns
Δ LF/HF	0.52 ± 1.37	0.33 ± 1.26	ns
15-min daily activity period			
Δ VLF (ms^2^)	98.95 ± 690.60	− 521.01 ± 1429.46	ns
Δ LF (ms^2^)	79.46 ± 227.56	− 136.14 ± 558.51	ns
Δ HF (ms^2^)	− 3.62 ± 240.60	− 94.00 ± 206.45	ns
Δ VHF (ms^2^)	1.35 ± 17.62	− 18.16 ± 42.83	ns
Δ LF/HF	0.45 ± 3.48	1.86 ± 4.34	ns
15-min N3 sleep period			
Δ VLF (ms^2^)	17.95 ± 1285.74	− 1900.12 ± 6942.25	ns
Δ LF (ms^2^)	128.03 ± 1178.53	− 575.56 ± 3243.54	ns
Δ HF (ms^2^)	− 80.29 ± 887.76	− 319.28 ± 705.22	ns
Δ VHF (ms^2^)	− 0.98 ± 66.36	− 28.81 ± 88.46	ns
Δ LF/HF	2.09 ± 3.58	− 1.40 ± 9.80	ns

Δ = measurement after the initiation of CPAP—measurement before the initiation of CPAP (positive values—parameter increase, negative values—parameter decrease)

Statistical significance of changes in the time and spectral HRV analysis was assessed separately in the subgroup of patients with severe and less than severe OSA, Tables 7 and 8.

**Table 7:** Parameters of the time analysis of heart rate variability before and after the initiation of CPAP therapy in subgroups differing in the severity of obstructive sleep apnea

	AHI before the initiation of CPAP < 30 (*n* = 16)			AHI before the initiation of CPAP ≥ 30 (*n* = 21)		
	before the initiation of CPAP	after the initiation of CPAP	*p*	before the initiation of CPAP	after the initiation of CPAP	*p*
Entire day (6:00–6:00)						
mRR (ms)	890.51 ± 118.56	934.16 ± 126.39	< 0.05	885.58 ± 108.77	863.74 ± 112.85	ns
SDNN (ms)	111.88 ± 31.17	116.61 ± 26.35	ns	117.63 ± 36.90	115.63 ± 49.69	ns
rMSSD (ms)	26.28 ± 9.95	26.40 ± 10.71	ns	40.69 ± 25.50	27.42 ± 10.67	< 0.05
SDSD (ms)	19.26 ± 8.93	18.31 ± 7.61	ns	32.80 ± 25.53	21.13 ± 9.39	ns
pNN50 (%)	4.81 ± 5.37	6.36 ± 7.94	ns	10.03 ± 8.04	5.49 ± 4.93	< 0.05
Hours of daily activity (6:00–22:00)						
mRR (ms)	854.48 ± 122.57	887.88 ± 128.51	ns	844.47 ± 116.75	820.84 ± 112.32	ns
SDNN (ms)	105.07 ± 30.71	101.48 ± 26.26	ns	102.12 ± 32.49	95.00 ± 35.37	ns
rMSSD (ms)	24.45 ± 10.90	24.61 ± 9.85	ns	34.40 ± 24.50	24.32 ± 9.19	ns
SDSD (ms)	18.16 ± 9.52	16.93 ± 6.58	ns	27.56 ± 24.24	18.79 ± 8.26	ns
pNN50 (%)	3.81 ± 4.82	5.60 ± 7.13	ns	6.83 ± 7.99	3.81 ± 4.24	ns
Hours of night rest (22:00–6:00)						
mRR (ms)	954.66 ± 124.03	1012.26 ± 1254.38	< 0.05	954.31 ± 102.61	939.02 ± 137.95	ns
SDNN (ms)	82.36 ± 15.79	89.25 ± 21.56	ns	101.57 ± 33.13	94.09 ± 40.71	ns
rMSSD (ms)	28.57 ± 8.93	29.02 ± 12.64	ns	48.15 ± 28.33	31.48 ± 14.44	< 0.05
SDSD (ms)	20.00 ± 8.56	19.91 ± 9.32	ns	37.49 ± 28.10	23.23 ± 12.18	< 0.05
pNN50 (%)	6.38 ± 6.47	7.68 ± 9.95	ns	15.10 ± 11.12	8.39 ± 7.42	< 0.05
15-min daily activity period						
mRR (ms)	866.62 ± 131.65	901.53 ± 165.85	ns	843.19 ± 155.06	805.09 ± 140.77	ns
SDNN (ms)	62.34 ± 32.55	58.40 ± 25.63	ns	58.45 ± 24.95	49.42 ± 24.17	ns
rMSSD (ms)	21.69 ± 7.90	23.98 ± 10.81	ns	31.51 ± 27.77	19.62 ± 8.67	ns
SDSD (ms)	14.64 ± 5.56	15.85 ± 6.90	ns	18.88 ± 10.11	13.72 ± 6.75	< 0.05
pNN50 (%)	3.03 ± 3.82	5.56 ± 7.24	ns	10.70 ± 21.70	2.54 ± 4.78	ns
15-min N3 sleep period						
mRR (ms)	955.00 ± 139.05	1005.97 ± 119.53	ns	967.85 ± 128.56	958.20 ± 154.87	ns
SDNN (ms)	43.01 ± 23.24	61.43 ± 36.03	< 0.05	64.11 ± 22.16	54.43 ± 31.63	ns
rMSSD (ms)	29.07 ± 16.83	27.21 ± 11.44	ns	45.88 ± 22.27	31.23 ± 16.01	< 0.05
SDSD (ms)	20.33 ± 15.70	18.13 ± 7.53	ns	33.41 ± 21.59	21.92 ± 12.63	< 0.05
pNN50 (%)	6.38 ± 11.43	6.80 ± 10.16	ns	15.57 ± 13.55	9.34 ± 9.30	< 0.05

**Table 8:** Parameters of the spectral analysis of heart rate variability before and after the initiation of CPAP therapy in subgroups differing in the severity of obstructive sleep apnea

	AHI before the initiation of CPAP < 30 (*n* = 16)			AHI before the initiation of CPAP ≥ 30 (*n* = 21)		
	before the initiation of CPAP	after the initiation of CPAP	*p*	before the initiation of CPAP	after the initiation of CPAP	*p*
Entire day (6:00–6:00)						
VLF (ms^2^)	926.38 ± 636.02	788.06 ± 441.87	ns	17,380.40 ± 73,100.64	912.55 ± 791.23	ns
LF (ms^2^)	576.87 ± 419.46	522.27 ± 469.73	ns	2158.26 ± 6248.85	882.45 ± 1178.43	ns
HF (ms^2^)	284.23 ± 231.72	262.20 ± 250.72	ns	726.62 ± 1060.47	372.08 ± 421.98	ns
VHF (ms^2^)	51.27 ± 58.95	40.37 ± 58.55	ns	90.95 ± 91.65	78.16 ± 144.70	ns
LF/HF	2.40 ± 1,42	2.92 ± 2.24	ns	2.04 ± 1.28	2.37 ± 2.11	ns
15-min daily activity period						
VLF (ms^2^)	656.73 ± 517.18	755.69 ± 867.18	ns	923.03 ± 1381.59	402.02 ± 327.94	ns
LF (ms^2^)	344.30 ± 272.98	423.76 ± 435.21	ns	448.39 ± 468.88	312.25 ± 386.96	ns
HF (ms^2^)	189.52 ± 198.32	185.90 ± 201.52	ns	197.83 ± 180.68	103.83 ± 132.35	< 0.05
VHF (ms^2^)	24.20 ± 15.22	25.55 ± 26.97	ns	51.48 ± 50.97	32.87 ± 35.89	ns
LF/HF	3.38 ± 2.71	3.83 ± 2.56	ns	2.81 ± 2.24	4.67 ± 4.38	< 0.05
15-min N3 sleep period						
VLF (ms^2^)	732.25 ± 1059.99	750.21 ± 687.37	ns	2703.05 ± 6760.18	802.94 ± 893.24	ns
LF (ms^2^)	474.08 ± 742.83	602.11 ± 836.51	ns	1247.04 ± 3063.44	671.48 ± 1028.33	ns
HF (ms^2^)	399.22 ± 711.39	318.93 ± 527.39	ns	695.27 ± 767.49	375.99 ± 351.68	< 0.05
VHF (ms^2^)	36.25 ± 46.40	35.27 ± 54.94	ns	88.73 ± 66.87	59.92 ± 94.28	ns
LF/HF	2.97 ± 1.93	3.06 ± 4.57	ns	4.37 ± 13.73	2.96 ± 4.85	ns

The time analysis in the subgroup of patients with less than severe OSA showed that initiation of CPAP therapy caused statistically significant increases in mRR parameter in the all-day and night measurements, as well as in the SDNN parameter measured in the 15-min N3 sleep period. In the subgroup of patients with severe OSA the initiation of CPAP therapy caused statistically significant decreases in rMSSD in the all-day, night rest and the 15-min N3 sleep period measurements; SDSD in measurements from night rest, 15-min daily activity and 15-min N3 sleep period and pNN50 in all-day, night rest and 15-min N3 sleep period measurements, Table 7.

Statistically significant changes in the spectral analysis of HRV parameters, after the initiation of CPAP, were observed only in the subgroup of patients with AHI ≥ 30. In this group, a significant decrease in HF was shown in the measurements from the 15-min daily activity period and the 15-min N3 sleep period, and a significant increase in the LF/HF ratio in measurements from the 15-min daily activity period, Table 8.

Correlation analysis showed negative linear relationships between the CPAP therapy parameters and changes in the parameters of time and spectral HRV analysis. A negative correlation were observed between the maximum positive airway pressure (PAP) value during the treatment and the change in rMSSD (r = − 0.37, *p* < 0.05) and SDSD (r = − 0.37, *p* < 0.05) parameters in all-day measurements. For the hours of daily activity and hours of night rest, there were also negative correlations between the rMSSD and SDSD parameters and the maximum PAP (r = − 0.36, *p* < 0.05, for both correlations). The mRR and the median PAP were negatively correlated during the day (r = − 0.33, *p* < 0.05), whereas the maximum PAP and SDNN were negatively correlated during the time of night rest (r = − 0.36, *p* < 0.05).

In terms of spectral HRV analysis, a negative correlation between the maximum PAP and VLF in the all-day measurement was observed (r = − 0.32, *p* < 0.05). In addition, in the 15-min daily activity period, there was a negative correlation between the mean PAP and median PAP and the change in LF (r = − 0.39, *p* < 0.05 and r = − 0.38, *p* < 0.05). In the 15-min N3 sleep period, there was a negative correlation between both the maximum PAP and the change in VLF and LF (r = − 0.39, *p* < 0.05 and r = -0.36, p < 0.05), and between the median PAP and the change in the HF parameter (r = − 0.33, *p* < 0.05).

## Discussion

The association of sleep apnea with heart rate variability was reported many years ago. Among others, lower SDNN values for the all-day measurements and the 15-min daily activity periods, as well as lower pNN50 values for the all-day measurements were described in OSA patients. Moreover, in patients with moderate OSA, lower values of the time analysis of HRV parameters were reported—especially for the 15-min daily activity periods and the parameters analyzed within them: SDNN, rMSSD, SDSD, pNN50. These HRV changes are caused by the increased activation of the sympathetic nervous system among OSA patients [10].

Sleep apnea is known to be associated with an increased risk of cardiovascular incidents, such as stroke, myocardial infarction, atrial fibrillation, or arterial hypertension [11–14]. This is due to recurrent episodes of hypoxia, resulting in endothelial dysfunction, increased vascular stiffness, inflammation within blood vessels, excessive blood clotting, changes in heart rhythm, increased activation of the sympathetic nervous system, as well as excessively negative chest pressure values observed during apnea episodes [10, 15–17].

Currently, the main non-invasive method of OSA treatment is CPAP therapy [18].

In a study by Sullivan et al., the positive effect of nasal CPAP (nCPAP) therapy on the reduction of 24-h blood pressure in patients with moderate to severe OSA was confirmed [19]. Similar to our study, an improvement in polysomnographic parameters such as AHI, blood saturation after the therapy, as well as the Epworth's scale score was reported. In many studies, CPAP therapy was also found to reduce the respiratory disturbance index (RDI), increase blood saturation during sleep and prolong sleep latency [20–22]. In our study, we observed an improvement in the mean and minimum saturation after treatment, a decrease in the ODI, and a more than five fold reduction in the AHI. In addition, sleep latency was extended and sleep efficiency increased, in short, an improvement in sleep parameters was observed.

The analysis of our results showed a statistically significant reduction in some HRV time analysis parameters after the initiation of CPAP therapy for the entire study group. In more detail, the rMSSD during the hours of night rest and 15-min N3 sleep period, as well as pNN50 during the night rest were reduced.

In the aspect of positive expected changes towards parasympathetic tone only in patients with AHI < 30 the increase in SDNN was observed 15-min N3 sleep period.

In terms of the time analysis, even before the initiation of CPAP therapy, there were differences between people with severe (AHI > 30) and milder forms of apnea (AHI < 30). Initially, rMSSD was higher in patients with AHI > 30, and the CPAP treatment caused a significant decrease in this parameter (during the entire day, during the hours of night rest, and in the N3 sleep period), again, it could be noticed that more significant changes concerned severely ill patients. Similarly, the pNN50 parameter decreased significantly after the treatment in patients with AHI > 30. In the group with AHI < 30, only an increase in the mRR parameter for the entire day and the hours of night rest was observed. Analysis of the correlation between CPAP variables and the HRV time analysis parameters showed an inverse correlation between the maximum PAP and the rMSSD parameter during many of the periods studied (entire day, the hours of daily activity, the hours of night rest, 15-min N3 sleep period). A similar correlation also occurred between the maximum PAP and the SDSD parameter. Moreover, in patients with AHI > 30, this parameter was significantly reduced after treatment.

Martin et al. reported, that untreated OSA significantly increased the risk of fatal cardiovascular events, while the implementation of CPAP therapy reduced the risk [23]. In another study, one-month CPAP therapy caused a significant reduction in AHI, an increase in the minimum blood saturation, as well as a decrease in the level of norepinephrine in the urine and the incidence of premature ventricular beats during sleep [24].

The benefits of CPAP therapy in OSA, concerning the duration of its use, have been reported by many authors. Among others, a reduction in blood pressure and mean heart rate values, as well as an improvement in left ventricular ejection fraction and sensitivity of baroreceptors were observed after one month of CPAP [25]. After 6 months of CPAP therapy for severe OSA, echocardiographic improvement in morphology and hemodynamic function of the myocardium was reported. Moreover, another study indicated that after 3 months of CPAP treatment the number of supraventricular and ventricular arrhythmias, including atrial fibrillation and sinus pauses, was significantly reduced [26, 27]

Our study showed no significant changes in the HRV spectral analysis parameters assessed for the entire study group, however, a decrease in HRV expressed for the group with AHI > 30 was observed. In more detail, a decrease in the HF value and, therefore, an increase in the LF/HF index, which is an astonishing shift rather not promoting a parasympathetic system tone. Theoretically, it can be concluded that the patients at a very early time after introducing CPAP therapy may have at first a paradoxical reaction observed, especially in some periods including night periods. The higher LF/HF ratio may indicate sympathetic dominance, which may happen when engaging in fight behaviors. It may be the early response to therapy, which probably could change after a longer period.

In former studies conducted by Karasulu et al. in HRV time analysis, there were no differences observed after initiation of the therapy after a maximally week period. Authors have found some positive significant changes in the aspect of spectral/frequency analysis using CPAP in LF, nuLF, nuHF (increased), and LF/HF (decreased) parameters [20]. In studies by Gilman et al. in the group of 19 patients with heart failure authors also proved that HRV parameter improved (there was an increase in HF) indicating improved vagal modulation of the heart rate after starting CPAP therapy, however only during morning wakefulness after a month of randomization [28]. Our study is supported by a little bigger group of patients, and, simultaneously, applies numerous sub-analyses taking into account different periods of the day and night, and also subgroups of patients.

The other studies by Grau et al. were carried out after a longer time after initiation of CPAP therapy, that is, after a year of treatment, in a group of 26 individuals and it has been observed that CPAP therapy only partially improved heart rate variability, exclusively during waking hours, simultaneously reducing episodes of atrial tachycardia [29]. Namely, rMSSD parameter improved and in patients > 50 years old LF and HF parameters from spectral analysis increased. The results are rather contradictory to our study where rMSSD decreased in patients with AHI > 30, for night hours and for a 24-h period and for the other subgroup and periods there were no significant differences. As it was abovementioned, in our results we noted the decrease in HF, among others, during N3 sleep period. HF components are predominantly modulated by the parasympathetic nervous system, so it was not a beneficial change. The same was for the 15-min daily activity period 15-min daily activity period LF/HF ratio was paradoxically higher.

Efazati et al. evaluated the changes in HRV one day after introducing CPAP therapy in 55 patients and they observed the improvement in autonomic balance expressed by changes in the low-frequency index, as well as increased high-frequency index and the significant decrease in LF/HF frequency ratio [30], which was different from our results. The authors of the study concentrated on the before–after study analyzing and the recording from night hours. In our studies we applied a more sophisticated survey considering different periods of night and day activities and the unique evaluation of the selected sleep period, that is N3 phase.

The influence of CPAP therapy on blood pressure values has been reported in many studies, including meta-analyses. Most of the results show that the use of CPAP therapy significantly lowers blood pressure, however, the effectiveness of the therapy differs depending on the patient's characteristics [31–33]. Younger patients with increased daytime sleepiness and more severe apnea, as well as those who use the CPAP device for longer periods during the night, seem to benefit more from the therapy. The improvement in cardiovascular parameters is likely due to an improvement in sympathetic/parasympathetic balance.

It is also worth mentioning the results of the meta-analysis published in 2014. This study indicated that CPAP therapy used in mildly symptomatic patients did not affect blood pressure, except for cases where it was used for more than 4 h per day, which resulted in a slight reduction in diastolic pressure [34]. There are also reports on the beneficial effects of adding CPAP therapy to antihypertensive pharmacotherapy. The use of CPAP therapy combined with losartan did not result in a significant reduction of mean blood pressure values during the day, however, it resulted in a significant reduction in systolic blood pressure at night and in the morning [35].

The benefits of CPAP therapy are numerous. It reduces hypoxia, lowers the day–night activity of the sympathetic nervous system, and reduced the number of atrial fibrillation episodes; however, the former data have been recently widely discussed [36]. The effect of CPAP therapy on the cardiac muscle was also assessed in terms of the reduction of arrhythmias and conduction disorders. Its use has been shown to reduce or eliminate premature ventricular beats, ventricular and supraventricular tachycardias, bradycardia, and atrioventricular conduction disorders [37–40]. Moreover, another study carried out on OSA patients aged between 58 and 64 years, showed that the incidence of supraventricular and ventricular arrhythmias was reduced after 3 months of CPAP therapy, and the occurrence of cardiac arrhythmias was correlated with the severity of OSA [27]. At the beginning of the study, 18.3% of patients suffered from atrial fibrillation, however, the percentage dropped to 8.6% after the initiation of CPAP therapy [27]. Abe et al. reported a significant decrease in atrial fibrillation and other atrial arrhythmias incidence, from 14 to 4%, as a result of CPAP therapy [41]. In 2015, a meta-analysis showed that patients with OSA treated with CPAP have a 42% lower risk of atrial fibrillation than untreated patients [42]. Moreover, in patients with diagnosed atrial fibrillation, the initiation of CPAP therapy decreased the likelihood of the transformation of paroxysmal arrhythmia into permanent [43]. CPAP is also useful in preventing atrial fibrillation recurrences after non-pharmacological interventions; it has been shown to reduce the number of relapses after both ablation and cardioversion [44–46]. However, even though numerous data have shown the benefits from CPAP therapy in the aspect of arrhythmia, a new randomized study from 2021 showed that CPAP may not be that efficient, indeed [47]. In a group of 579 patients with moderate to severe disease (apnea–hypopnea index ⩾15) and paroxysmal atrial fibrillation, the treatment with CPAP did not result in a statistically significant reduction in the burden of AF. Thus, still this problem remains controversial.

In general, atrial arrhythmias occur most frequently in patients with moderate to severe OSA [48] and it is known that CPAP therapy is most effective in these groups of patients and can reduce the incidence of arrhythmias [49]. Supraventricular arrhythmias and ventricular accessory beats may occur in up to 66% of patients with OSA and typically occur in patients with advanced sleep apnea. It is believed that it may be, among others, the effect of disturbed balance in the vegetative system [27].

Some studies show an association between sleep apnea and serious cardiovascular events, including fatal ones. The relative risk of sudden cardiac death in OSA patients, especially untreated patients, is higher than in the general population [23, 50]. It is caused by the influence of hypoxia on the induction of inflammatory reaction (activation of the transcription of pro-inflammatory factors: CRP, IL-6, TNF-a) and also by activation of the sympathetic system and the induction of oxidative stress, which affects the functioning of the circulatory system [51, 52].

Up to 50% of patients with OSA may suffer from various types of arrhythmias, which include almost all types of supraventricular and ventricular arrhythmias [53]. This may be a factor in increasing mortality in this group of patients [54]. In a study conducted in 2020, the length of sleep apnea correlated positively with the amount of ultra-short-term HRV [55].

There are some limitations of the study. One of them may be the small number of patients, however, in this type of research study numbers are not very high even in tertiary level hospitals and clinics. Generally, the results require confirmation in further long-term studies on a vast group of patients, Also, when comparing with other researches in this field in different studies there have been various periods between introducing CPAP therapy and the second test ranging from 1 to 2 days, even to one year, that is why the available data from medical reports are not fully comparable.

## Conclusions

1. The initiation of CPAP therapy in patients with OSA not only improves sleep characteristics but also affects heart rate variability.
2. In the majority of parameters after a month of therapy with CPAP there were no significant differences in time and frequency HRV analysis.
3. The response to CPAP therapy may depends on the severity of the apnea. In patients with AHI > 30, a reduction in HRV was observed both in the time and spectral analysis in some parameters, while in patients with AHI < 30, an increase in SDNN parameter from time analysis was observed.

## Data Availability

The data are not publicly available; they may be made available upon a justified request addressed to the corresponding author.
